# Azithromycin and cough-specific health status in patients with chronic obstructive pulmonary disease and chronic cough: a randomised controlled trial

**DOI:** 10.1186/1465-9921-14-125

**Published:** 2013-11-14

**Authors:** Farida F Berkhof, Nynke E Doornewaard-ten Hertog, Steven M Uil, Huib AM Kerstjens, Jan WK van den Berg

**Affiliations:** 1 Department of pulmonary diseases, Isala, Dr. van Heesweg 2, 8025AB, Zwolle, the Netherlands; 2 Department of pulmonary diseases, University of Groningen, University Medical Center, Hanzeplein 1, Groningen 9713 GZ Groningen, the Netherlands

**Keywords:** COPD, Health status, Azithromycin, LCQ, Cough

## Abstract

**Background:**

Macrolides reduce exacerbations in patients with COPD. Their effects on health status has not been assessed as primary outcome and is less clear. This study assessed the effects of prophylactic azithromycin on cough-specific health status in COPD-patients with chronic productive cough.

**Methods:**

In this randomised controlled trial 84 patients met the eligibility criteria: age of ≥40 years, COPD GOLD stage ≥2 and chronic productive cough. The intervention-group (n = 42) received azithromycin 250 mg 3 times a week and the control-group (n = 42) received a placebo. Primary outcome was cough-specific health status at 12 weeks, measured with the Leicester Cough Questionnaire (LCQ). Secondary outcomes included generic and COPD-specific health status and exacerbations. Changes in adverse events and microbiology were monitored.

**Results:**

Mean age of participants was 68 ± 10 years and mean FEV1 was 1.36 ± 0.47 L. The improvement in LCQ total score at 12 weeks was significantly greater with azithromycin (difference 1.3 ± 0.5, 95% CI 0.3;2.3, p = 0.01) and met the minimal clinically important difference. Similar results were found for the domain scores, and COPD-specific and generic health status questionnaires. Other secondary endpoints were non-significant. No imbalances in adverse events were found.

**Conclusions:**

Prophylactic azithromycin improved cough-specific health status in COPD-patients with chronic productive cough to a clinically relevant degree.

**Trial registration:**

ClinicalTrials.gov NCT01071161

## Background

Chronic obstructive pulmonary disease (COPD) is one of the leading causes of death [1], with an estimated worldwide prevalence of up to 10.1% [2] and it is expected to increase over the coming decades [3]. Important and common symptoms in patients with COPD are chronic cough and sputum production, or chronic bronchitis [4]. Additionally, approximately up to 50% of patients with moderate to severe COPD have bronchiectasis at least to some degree and this is associated with a poorer prognosis [5,6]. Chronic cough and sputum production are caused by inflammation due to smoking or inhaled other irritants [7]. Mucus hypersecretion by itself facilitates bacterial proliferation and colonization which in turn contributes to chronic inflammation [8,9]. Chronic obstructive bronchitis, i.e. COPD, is associated with progressive lung function loss, more frequent exacerbations, and hospitalisations [8]. The latter lead to a deterioration of health status [10]. Improving health status is an important goal in the treatment of COPD patients [3]. Inhaled glucocorticoids, long-acting beta_2_-agonists, and long-acting anticholinergics have all been shown to reduce exacerbation frequency in COPD, but despite these therapies, the average frequency of acute exacerbations still remains approximately 1.4 each year [11]. An addition to the usual therapy is long-term macrolide use, of which the mechanism of action is attributed to the immunomodulatory effects as well as to diverse actions that suppress microbial virulence factors beyond their antibacterial effects [12-14]. In several studies [15-21] macrolides have been demonstrated to reduce the frequency of COPD exacerbations of which four studies [16,18,19,21] examined disease specific and generic health status as a secondary outcome only. None of these studies addressed cough-specific health status specifically. We were interested in cough because it is very relevant to patients’ daily life and chronic cough and sputum production are also risk factors for worse outcomes in COPD patients [22]. The impact on cough-specific health status in these patients is largely unknown. The Leicester Cough Questionnaire (LCQ) is a cough-specific health status questionnaire which is originally validated for a population of general patients presenting with chronic cough [23]. Recently, the LCQ was validated to measure cough-specific health status in patients with COPD and chronic bronchitis [24].

Hence, the primary hypothesis was that prophylactic azithromycin improves cough-specific health status in patients with COPD and chronic productive cough. Important secondary hypotheses were that it also leads to improvements in generic and COPD-specific health status.

## Methods

### Study design

The study was designed as a single-centre parallel group randomised double-blind placebo controlled trial. It was carried out in the Isala klinieken, a large teaching hospital in Zwolle, the Netherlands. Approval of the local ethics committee was received (NL19886.075.07, local number: 07.0971) and the study was registered at ClinicalTrials.gov (NCT01071161). All participants provided written informed consent.

### Participants

Eligible patients were ≥ 40 years, had a clinical diagnosis of COPD GOLD stage ≥2 (defined as a post bronchodilator of forced expiratory volume in 1 second (FEV_1_) <80% and a ratio of FEV_1_ to forced vital capacity of <70%), and were suffering from chronic productive cough, defined as cough for at least the last 12 weeks, in two subsequent years. Exclusion criteria were a prior history of asthma; use of intravenous or oral corticosteroids and/or antibiotics for an exacerbation three weeks before inclusion; other relevant lung or liver diseases at the discretion of the treating physician; pregnancy or lactation; use of macrolides in the last six weeks prior to inclusion; allergy or intolerance to macrolides; or use of other investigational medication started two months prior to inclusion.

Long term treatment with aerosolized antibiotics, inhaled corticosteroids, and/or bronchodilators was permitted during the trial, provided that it was kept constant.

### Randomisation and blinding

Patients were randomly assigned, without stratification, to receive azithromycin 250 mg three times a week or an identical appearing placebo for 12 weeks. Randomisation codes were generated using a computer allocation program, with a 1:1 ratio and a permutated block size of 4. Investigators, research nurses, and participants were masked to treatment allocation until final analyses of the data were performed.

### Procedures

Patients were instructed to take the study medication weekly on Monday, Wednesday, and Friday. Study medication was prepared by Central Hospital Pharmacy, The Hague, the Netherlands and was distributed by our hospital pharmacy. During the first outpatient visit, baseline spirometry, smoking status, pulmonary medication, and laboratory blood values (aspartate transaminase (ASAT), alanine aminotransferase (ALAT), and C-reactive protein (CRP)), and a spontaneous sputum sample for culture of respiratory pathogens were collected. Patients were asked to complete the LCQ [23,24], SGRQ [25], and SF-36 [26], to assess cough-specific, disease-specific (COPD), and generic health status, respectively. At two, six, nine, and eighteen weeks, telephone calls were scheduled to collect data on adverse events, concomitant medication, and to ask the patient to complete the LCQ and return it by mail. At 12 weeks a second outpatient visit was planned at which spirometry was done and blood laboratory values, and a spontaneous sputum sample were collected. Also, the LCQ, SGRQ, and SF-36 were repeated. Adherence was assessed by counting the unused pills. All participants were analysed for bronchiectasis by high resolution CT-thorax at baseline. Criteria for the diagnosis of bronchiectasis were lack of tapering, visibility of bronchi within 1 cm of the pleura and bronchial dilatation (bronchial diameter larger than that of the accompanying pulmonary artery while avoiding slices close to bronchial bifurcations) [6].

### Endpoints

The primary endpoints were mean LCQ total and domain scores at 12 weeks. The LCQ total scores vary from 3 to 21 and the domain scores vary from 1 to 7, with a higher score signifying better health status, the MCID is 1.3 [23,24]. The secondary endpoints at 12 weeks were: St. George’s Respiratory Questionnaire (SGRQ) total score (range from 0-100, a low score indicates a good health status, the MCID is 4 [25,27]), Short Form 36 (SF-36) score (range from 0-100, higher scores represent better health status, the MCID is 4 [26,28,29]), post-bronchodilator spirometry (FEV_1_, FEV_1_%predicted), blood values, and microbiology. Other endpoints included time to first exacerbation of COPD, defined as a sustained worsening of the patient’s condition, from the stable state and beyond normal day-to-day variations, that necessitates treatment with prednisolone, antibiotics or a combination of both [30], as well as exacerbation and hospitalization rates for COPD, and adverse events, during 18 weeks.

### Sample size considerations

Sample size calculation was based on LCQ total scores. At the time of designing the study no MCID estimate of the LCQ was available. Therefore, a difference between the study groups in mean LCQ total score of at least 1.5 (SD = 2.0) points at 12 weeks was chosen. To be able to demonstrate this difference with a power of 90% and a two-sided α level of 0.05, 42 patients were needed in each group (taking into account a drop-out rate of 10%).

### Statistical analyses

Primary and secondary analyses were done according to the intention-to-treat principle. Missing LCQ data at 12 weeks were imputed using the last observation carried forward, from 9 weeks, when possible. Normal distributions of outcomes were checked using histograms. Baseline characteristics, microbiology outcomes, and blood values at baseline and at 12 weeks were examined with descriptive statistics. Differences in primary and continuous secondary outcomes (i.e. SGRQ scores, SF-36 scores, and spirometry) were tested using ANCOVA, adjusting for baseline values. Interaction terms between treatment group and baseline value were checked to explore whether the extent of treatment response varied dependent on the value of the baseline value. A log-rank test was used to test differences in time to first exacerbation between study groups which were graphically presented by Kaplan-Meier curves. Between-group comparisons of proportions were performed using Chi-squared tests. P-values <0.05 were considered significant. Analyses were performed using SPSS-Statistics version 19.0 (IBM corporation, Armonk, NY, USA).

## Results

### Participants

**Figure 1:**
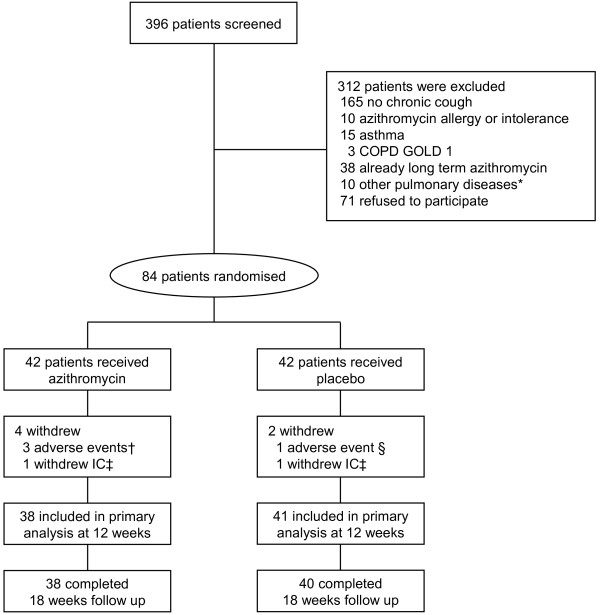
**Consort flow chart.** * 5 patients with lung cancer, 4 patients with idiopathic interstitial lung disease, 1 patient with bronchiectasis † 2 patients with diarrhoea and 1 with disturbance of taste. ‡ informed consent. § patient with disturbance of taste. Withdrew after 12 weeks.

Recruitment started Sept. 15, 2009 and ended Oct. 14, 2011, and the last patient finished after 18 weeks of follow up. In total 84 patients were randomised. Screening, randomisation, follow up, and losses after randomisation are shown in a consort flow chart, Figure 1. Baseline characteristics were similar between the groups, except for a small difference in proportion of patients using inhaled corticosteroids and the LCQ total score, Table 1. Adherence with study medication was high during the study. On average, 85% and 92% of the patients used all weekly dosages in the azithromycin and placebo groups, respectively (p = 0.48 for difference).

**Table 1:** Patient characteristics

		**Azithromycin (n = 42)**	**Placebo (n = 42)**
Age (years), mean (SD)		67 (9)	68 (10)
Male sex, n (%)		31 (74)	32 (76)
FEV_1_ (L), mean (SD)		1.41 (0.52)	1.32 (0.42)
FEV_1_ %predicted, mean (SD)		49.8 (16.4)	47.4 (12.9)
FEV_1_/FVC ratio (%),mean (SD)		42.2 (11.9)	43.2 (11.7)
BMI (kg/m^2^), mean (SD)		27.2 (4.3)	25.7 (5.8)
Pack years, median (range)		30.5 (3-110)	30.0 (1-69)
Current smoker, n (%)		14 (33)	15 (36)
Blood values,	CRP (mg/L), median (range)	6.5 (0-46)	4.0 (0-25)
	ASAT (U/L)* mean (SD)	24.2 (6.5)	26.4 (9.8)
	ALAT (U/L)* mean (SD)	24.4 (8.0)	24.4 (13.7)
LCQ scores, mean (SD)	Total	14.5 (2.3)	13.4 (3.3)
	Physical	4.3 (0.7)	4.2 (1.0)
	Psychological	5.1 (1.0)	4.7 (1.1)
	Social	5.0 (1.1)	4.5 (1.5)
Bronchiectasis, n (%)		18 (42.9)	16 (38.1)
Inhaled medication, n (%)	Long acting beta_2_ agonists	34 (81.0)	35 (83.3)
	Long acting anticholinergics	27 (64.3)	24 (57.1)
	Corticosteroids	41 (98.0)	35 (83.0)
Number of exacerbations in previous year, median (range)		1 (0-8)	1 (0-13)

FEV_1,_ forced expiratory volume in 1 s; FVC, forced vital capacity; BMI, body mass index; CRP, C-reactive protein; ASAT, aspartate transaminase; ALAT, alanine aminotransferase; LCQ, Leicester Cough Questionnaire. Total scores range from 3-21 and domain scores from 1-7. Higher scores signify better health status.

* In ASAT and ALAT n = 41.

### Primary outcome

A significantly greater mean increase in LCQ total score after 12 weeks was found in the azithromycin group compared with placebo, 1.3 ± 0.5 (95% CI 0.3; 2.3, p = 0.01). Significant differences were also found for the different domain scores of the LCQ, Table 2.

**Table 2:** Change in LCQ scores after 12 weeks adjusted for baseline values

	**Azithromycin (n = 38)**	**Placebo (n = 41)**	**Difference**	**95% CI**	**p Value**
Total	2.2 ± 0.4	0.9 ± 0.3	1.3 ± 0.5	0.3;2.3	0.01
Physical	0.6 ± 0.1	0.2 ± 0.1	0.4 ± 0.2	0.1;0.8	0.01
Psychological	0.8 ± 0.1	0.3 ± 0.1	0.5 ± 0.2	0.2;0.9	0.006
Social	0.8 ± 0.2	0.4 ± 0.2	0.4 ± 0.2	0.01;0.9	0.046

All scores are presented as mean with standard error.

LCQ, Leicester Cough Questionnaire. Higher scores signify better health status. A change of 1.3 points is regarded as minimal clinically important.

**Figure 2:**
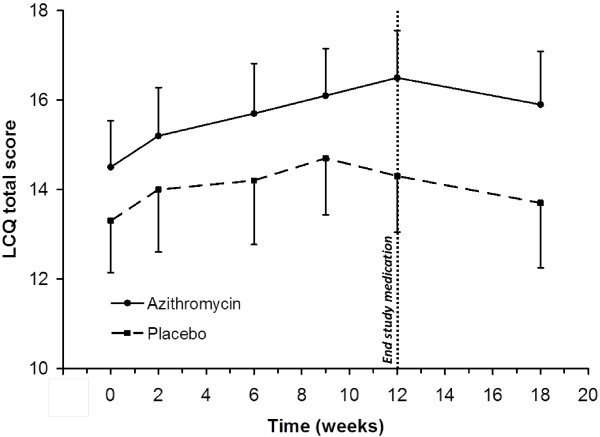
**Change over time in LCQ total score.** Repeated measures of the Leicester Cough Questionnaire (LCQ) total scores at 0, 2, 6, 9, 12 and 18 weeks between the azithromycin (n = 38) and placebo (n = 39) group. Error bars indicate 95% confidence intervals.

Repeated measurements analysis over the full treatment period also showed that the mean differences between groups for the LCQ total score were significant (p = 0.01), in favour of the azithromycin group (Figure 2). According to the study protocol prophylactic azithromycin was stopped at 12 weeks which resulted in a decrease of the LCQ total score in the azithromycin group, whereas in the control group this decrease already started at 9 weeks.

As described in the methods, the interaction term between study group and baseline value was checked in the ANCOVA analysis. In all models including LCQ total and domain scores, significant interaction was present. In other words, treatment responses varied dependent on the value of the baseline LCQ total score. To investigate the impact of interaction the study population was divided according to the median of baseline LCQ total score which was 14.1, since no meaningful cut-off values have so far been proposed in literature. The improvement with azithromycin compared to placebo in total LCQ score in the complete population proved to be due almost entirely to the patients with a low LCQ total score (<14.1). The difference over 12 weeks for the azithromycin group with a low LCQ total score at baseline was 2.6 ± 0.8 (95% CI 1.0;4.2, p = 0.002) and for the azithromycin group with a high LCQ total score at baseline (≥ 14.1) was 0.1 ± 0.6 (95% CI -1.1;1.2, p = 0.90).

### Secondary outcomes

#### SGRQ and SF-36

The improvement in SGRQ total score over 12 weeks was greater with azithromycin than with placebo: mean difference was -7.4 ± 2.5 (95% CI -12.5; -2.5 p = 0.004). The improvements in the SGRQ domain scores symptoms and impact were also significant, Table 3. Similar to the primary outcome the improvements in SGRQ scores also proved to be due almost entirely to the patients with a low LCQ total baseline score (<14.1). The difference of the SGRQ total score after 12 weeks in patients with a low LCQ total baseline score was -13.8 ± 4.1 (95% CI -22;-5.5 p = 0.002) in favour of the azithromycin group. On the contrary, the difference of the SGRQ total score for patients with a high LCQ baseline score was -1.6 ± 2.9 (95% CI -7.5;4.4 p = 0.59). Significant mean differences at 12 weeks in favour of the azithromycin group were found in the SF-36 scores: general health, role physical, social functioning, and mental health, see Table 3. Comparable with the primary and the SGRQ findings, patients with a low LCQ baseline score showed a greater difference after 12 weeks in the SF-36 domain general health (14.1 ± 5.0 (95% CI 4.0;24.3 p = 0.01)) in favour of the azithromycin group than patients with a high LCQ baseline score (2.4 ± 4.2 (95% CI -6.2;11.0 p = 0.57)). Analysis of the other SF-36 domain scores follows the same tendency, except for role physical.

**Table 3:** Change in SGRQ and SF-36 scores after 12 weeks adjusted for baseline values

	**Azithromycin**	**Placebo**	**Difference**	**95% CI**	**p Value**
**SGRQ**	(n = 37)	(n = 37)			
Total score	−6.6 ± 1.8	0.9 ±1.8	−7.5 ± 2.5	−12.5;-2.5	0.004
Symptoms	−9.2 ± 3.0	0.1 ± 3.0	−9.1 ± 4.2	−17.6;-.07	0.034
Activity	−4.1 ± 2.3	0.2 ± 2.3	−4.3 ± 3.2	−10.7;2.1	0.18
Impact	−7.3 ± 2.0	1.6 ± 2.0	−8.9 ± 2.8	−14.5;-3.3	0.002
**SF-36**	(n = 37)	(n = 37)			
General health*	4.5 ± 2.4	−3.8 ± 2.4	8.3 ± 3.4	1.6;15	0.016
Physical functioning	5.5 ± 2.2	0.7 ± 2.3*	4.8 ± 3.2	−1.5;11.1	0.13
Bodily pain	5.6 ± 3.3	−0.9 ± 3.3	6.5 ± 4.7	−2.9;15.9	0.17
Vitality*	4.0 ± 2.4	−2.0 ± 2.4	6.0 ± 3.4	−0.8;12.9	0.08
Role physical	16.2 ± 5.4	−1.1 ± 5.4	17.3 ± 7.6	2.2;32.5	0.025
Role emotional	−0.4 ± 5.6	−6.3 ± 5.6	5.9 ± 7.9	−9.8;21.7	0.46
Social functioning	4.4 ± 3.1	−8.5 ± 3.1	12.9 ± 4.4	4.0;21.7	0.005
Mental health*	2.2 ± 1.9	−3.5 ± 1.9	5.7 ± 2.7	0.4;11.0	0.037

All scores are presented as mean with standard error.

SGRQ, St. George’s respiratory questionnaire. Scores range from 0-100. A low score indicates a good health status, the minimal important difference is 4; SF-36, Short-form 36. Scores range from 0-100, higher scores represent better health status. The minimal important difference is 4.

*n = 36.

#### Exacerbations

A COPD exacerbation occurred in 10 (23.8%) patients in the azithromycin group and 17 (40.5%) in the placebo group, p = 0.10.

**Figure 3:**
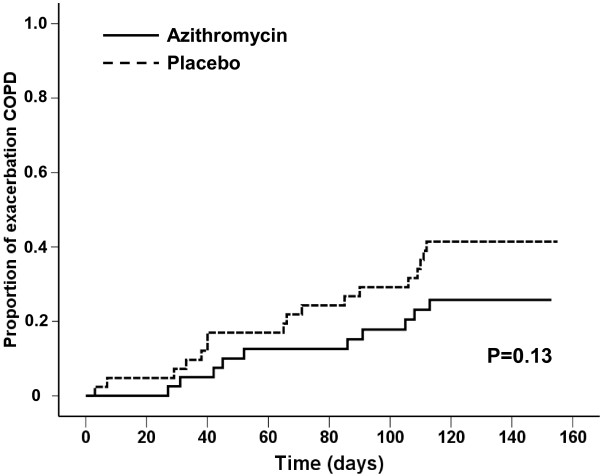
**Time to first exacerbation COPD.** Kaplan Meier curves showing the proportion of patients with a first exacerbation against time in days for the azithromycin (n = 42) and placebo (n = 42) group.

Because less than 25% of the patients in the azithromycin group had an exacerbation in 18 weeks the 20^th^ percentile time to the first exacerbation was calculated, which was 105 ± 30 days in participants receiving azithromycin compared with 66 ± 21 days in the placebo group (p = 0.13; log-rank test), Figure 3.

Four (9.5%) patients in the azithromycin group and 5 (11.9%) patients in the placebo group were hospitalized for COPD.

Analogous to the other outcomes there was a trend towards a lower exacerbation frequency in the patients with a low LCQ baseline total score which received azithromycin.

#### Spirometry, blood, sputum and adverse events

There were neither statistically significant nor clinically relevant differences in FEV_1_.

ASAT and ALAT were similar in both groups at baseline with no relevant changes in either group after 12 weeks. Furthermore, no individual changes above normal values in ASAT and ALAT were found.

A reduction of respiratory pathogens was seen in the azithromycin group after 12 weeks, Table 4.

**Table 4:** Microbiology

	**Azithromycin**		**Placebo**	
	**Baseline**	**12 weeks**	**baseline**	**12 weeks**
**Microbiology, n (%)**	(n = 40)	(n = 30)	(n = 41)	(n = 31)
*Streptococcus pneumoniae*	5 (11.9)	0 (0)	3 (7.1)	2 (4.8)
*Haemophilus influenzae*	11 (27.5)	4 (13.3)*	7 (17.1)	10 (32.3)
*Moraxella catarrhalis*	5 (12.5)	0 (0)	5 (12.2)	3 (9.7)
*Pseudomonas aeruginosa*	0 (0)	1 (3.3)	2 (4.9)	3 (9.7)
*Staphylococcus aureus*	1 (2.5)	0 (0)	1 (2.4)*	0 (0)

*one patient with azithromycin resistant bacteria.

Adverse events were comparable in both groups (Table 5). In the azithromycin group three patients with adverse events stopped using study medication, two patients had diarrhoea, and one patient had disturbance of taste. In the placebo group one patient stopped study medication because of disturbance of taste.

**Table 5:** Adverse events in 12 weeks

**Adverse events**	**Azithromycin**	**Placebo**	**p-value**
Gastro-intestinal*, n (%)	5 (11.9)	6 (14.3)	0.75
Upper respiratory †, n (%)	7 (16.7)	8 (19.0)	0.78
Cardiovascular ‡, n (%)	2 (4.8)	1 (2.4)	0.56
Other§, n (%)	3 (7.1)	5 (11.9)	0.71

*gastro-intestinal adverse events were diarrhoea, nausea and ulcus ventriculi.

†Upper respiratory adverse events were common cold, dyspnoea and cough.

‡Cardiovascular adverse events: myocardial infarction, supraventricular tachycardia, heart failure.

§Other include: pruritis, headache, disturbance of taste, malaise, atralgia and hyperhidrosis.

## Discussion

Our study is the first randomised placebo controlled trial to evaluate the effect of prophylactic azithromycin on cough-specific health status (LCQ) in COPD patients with chronic bronchitis. Cough-specific health status, as well as disease specific (SGRQ), and generic (SF-36) health status improved statistically significantly with azithromycin compared to placebo, with improvements equal to or exceeding the MCID. Moreover, there was a clear trend for azithromycin to increase the time to the first exacerbation compared to placebo. Adverse events were similar in both groups, which indicated azithromycin was well tolerated.

The beneficial effect of azithromycin was apparent for the study population as a group, but patients with a high baseline LCQ total score experienced no effects of azithromycin on cough-specific health status and the other efficacy outcomes at all. Although all patients recruited for this study met the predefined definition of chronic productive cough, it appears that the LCQ could discriminate between patients who respond to azithromycin and those who did not. Perhaps, COPD patients with chronic cough are more heterogeneous than expected, depending on the degree of impairment of cough-specific health status, the LCQ might discriminate between different types or severity of cough in COPD patients. It has been shown before that chronic cough with persistent symptoms has a larger impact on activities of daily life than morning cough or incidental cough [31].

Recent studies [15-21] assessing the effect of prophylactic antibiotics in patients with COPD and chronic bronchitis focused particularly on reducing exacerbations. Six of these studies used macrolides i.e. erythromycin, clarithromycin, and azithromycin respectively. These antibiotics belong to the same category, US FDA approved, and can thus be compared [32]. Four studies explored health status as a secondary outcome [16,18,19,21], of which one study did not include concurrent controls [18]. In these studies the disease specific and generic health status were measured as a secondary outcome only and findings were inconsistent. None of these studies addressed cough-specific health status specifically.

In one large clinical trial [16] the dose of azithromycin was 250 mg a day for one year, resulting in development of nasopharyngeal colonization with azithromycin-resistant pathogens, 81% versus 41% for the azithromycin group and the placebo group respectively. It has been suggested that the daily dose might be more than needed, especially given these resistance problems. In our study, with azithromycin 250 mg three times per week, only one patient developed an azithromycin-resistant *Haemophilus Influenzae*, although the follow up period was only three months. Our lower dose of azithromycin of 250 mg of three times a week seemed equally effective and sufficient.

Another important question is the optimal duration of treatment. In our study we chose to treat patients for 3 months. In 2 recent studies [16,33] patients were treated with prophylactic azithromycin for 6 months and for 1 year respectively. It is interesting to note that in both studies the largest effect was seen in the first 3 to 4 months, afterwards a more equal exacerbation rate was noticeable for both the azithromycin and the control group. Perhaps, an alternate treatment scheme of prophylactic azithromycin, e.g. every other 3 months, is preferable over continuous use, and thus preventing unnecessary treatment with long-term antibiotics, which has important consequences with respect to side effects, and bacterial resistance.

One obvious limitation of our study was the small group, though the study was sufficiently powered for the primary outcome, LCQ scores at 12 weeks. However, the primary outcome was missing in ten patients; in five cases data from nine weeks could be imputed which probably underestimated treatment response at 12 weeks. We chose to impute data with the last observation carried forward approach to increase power and precision, though there also limitations to this approach [34,35]. Another limitation is the MCID of the LCQ is not yet established in COPD patients with chronic cough; therefore the MCID in patients with chronic cough was used in this study. It will be clinically useful to determine a MCID of the LCQ specifically in COPD patients with chronic cough. Finally, since objective cough frequency does not always correlate with symptoms or cough-specific health status, the use of cough recorders at home to objectively assess cough would have been an interesting but costly adjunct to the study [36].

## Conclusions

In conclusion, this study showed that prophylactic azithromycin of 250 mg, three times a week for three months, provided significant and clinically relevant improvements in cough specific health status in patients with COPD and chronic productive cough. This was supported by improvements in disease specific and generic health status parameters, and although not powered to assess a reduction in exacerbation rate, the tendency was nevertheless clear. The effects were largely limited to those with a high burden of cough specific complaints at baseline. Interesting next steps would be studies limited to patients with a high LCQ, perhaps assessing also the level of airway inflammation. We believe it is an interesting thought to further elaborate on duration of macrolides treatment and whether it should be continuous or recurrent.

## Abbreviations

COPD: Chronic Obstructive Pulmonary Disease; LCQ: Leicester Cough Questionnaire; SGRQ: St. George’s Respiratory Questionnaire; MRF26: the Maugeri Respiratory Failure questionnaire; SF-36: Short Form 36; ASAT: aspartate transaminase and; ALAT: alanine aminotransferase; CRP: C-reactive protein; FEV1: forced expiratory volume in 1 second; FVC: forced vital capacity.

## Competing interests

This trial was supported by Teva Pharma, the Netherlands, which provided the azithromycin tablets.

## Authors’ contributions

JWKB and HAMK contributed to the study design. SU and FFB contributed to the statistical analysis. NED, SU, HAMK, JWKB and FB contributed to the interpretation of the results, as well as the manuscript drafting and revision. All authors read and approved the final manuscript.
